# Street Architecture

**Published:** 1900-11-24

**Authors:** 


### Street Architecture.

At a time when suggestions for "housing" the poor—suggestions mostly more or less irreconcilable with common sense and sound economy—form the stock-in-trade of innumerable political quacks and self-styled "progressive" reformers, it is very delightful to receive from an eminent engineer suggestions for the better housing of classes somewhat higher in the social scale, such as, presumably, would be the tenants of the new street which is about to be made between Holborn and the Strand. It is true that the tenants in question do not appear to have been the primary objects of Sir Frederick Bramwell's concern, and that he has been thinking mainly of the best methods of employing and covering the space which the London County Council will soon have at its disposal; but it is no less true that the former consideration must, to a very great extent, be involved in the latter, and that every construction which increases the general value of the houses, and the impressiveness and convenience of the street architecture, will be likely to increase, in the same proportions, the comfort and wholesomeness of the individual dwellings of which the street is composed. Sir Frederick Bramwell's proposal has been too fully discussed in the *Times* and in other papers for it to be necessary to enter here into any detailed description of it; and it is sufficient to say that he would make the ordinary ground-floor shops project beyond the general line of the houses of which they formed part, and carry upon their roofs a second or elevated footway upon which a "first-floor" row of shops would open, and which would be accessible from the footway below by stairs at convenient intervals, and be partly covered by the projection over it of the second floors of the buildings. Bridges would be carried over side streets at the level of the upper footway, and similar bridges would cross the main street with sufficient frequency. The suggestion, therefore, is that every house would have two shops, an upper and a lower one; that the footway in front of the upper shops would be to a great extent protected from rain or snow; and that the stairs and footbridges would allow pedestrians to cross every side street at the first-floor level, or to cross from one side of the main street to the other, without either arresting the carriage traffic or being in danger from it. Increased house value, increased convenience, diminution of mud, and protection from snow and rain are held out as the chief advantages to be expected from the adoption of the plan.

From a sanitary point of view the most important consideration arising out of Sir Frederick's proposal has relation to the probable cleanliness of the stairs and footway. This question has already been raised in the *Times*; and Sir Frederick replied to it, not very effectively, by saying that he had visited the stairs leading from the Holborn Viaduct to Farringdon Street, and had found them, if not all that could be desired, at least not very dirty. He would have done better to visit the stairs leading from the Thames Embankment either to Wellington Street or to the railway bridge at Charing Cross. The Farringdon Street stairs are scarcely used, except as playgrounds for a few stray children; the Embankment stairs are a good deal used. In winter they would furnish mud by shovelfuls; in summer, dust by handfuls. If the construction proposed were of such a nature that the stairs and footways could be effectually cleaned and washed with hose every night, and if this were done by the municipality as a regular part of its duty, some of the indicated advantages might, no doubt, be secured; but if the cleansing were left to the residents, to be accomplished in a fragmentary way by each householder, with such imperfect means as he could command, the stairs and footways would be filthy, and the dwellings giving upon them would soon become correspondingly unwholesome. This seems to us to be the real difficulty of the case, and the other objections which have been raised are in large measure imaginary. Mr. Frederic Harrison, for example, is filled with holy horror at the idea of the County Council letting its sites under conditions as to the class of building which should be erected—in such a way, for example, as to compel the adoption of Sir Frederick Bramwell's scheme. The idea of liberty entertained by some is based upon a perfect readiness to *re*strain, to say "you shall *not*," with a corresponding unwillingness to *con*strain, to say "you *shall*." True liberty, however, says you *shall* do everything that is clearly for the good of the community, and only says you shall *not* in those exceptional cases in which the proposed form of activity would be in the highest degree hurtful and injurious.

